# Incorporating an Increase in Plant-Based Food Choices into a Model of Culturally Responsive Care for Hispanic/Latino Children and Adults Who Are Overweight/Obese

**DOI:** 10.3390/ijerph17134849

**Published:** 2020-07-06

**Authors:** Pramil N. Singh, Jessica Steinbach, Anna Nelson, Wendy Shih, Mary D’Avila, Selene Castilla, Michael Jordan, William J. McCarthy, David Hayes-Bautista, Hector Flores

**Affiliations:** 1 Center for Health Research, Loma Linda University, Loma Linda, CA 92350, USA; jsteinbach@llu.edu; 2 School of Public Health, Loma Linda University, Loma Linda, CA 92350, USA; anelson@llu.edu (A.N.); wshih@llu.edu (W.S.); 3 Diabetes Education Center, Adventist Health White Memorial Medical Center, Los Angeles, CA 90033, USA; DAvilaME@ah.org (M.D.); CastilS3@ah.org (S.C.); 4 Research and Center for Hispanic Health, Adventist Health White Memorial Medical Center, Los Angeles, CA 90033, USA; JordanMB@ah.org; 5 Department of Health Policy and Management, School of Public Health, UCLA Jonsson Comprehensive Cancer Center, Los Angeles, CA 90095, USA; wmccarth@ucla.edu; 6 Center for Study of Latino Health and Culture, David Geffen School of Medicine, University of California at Los Angeles, Los Angeles, CA 90095, USA; dhayesb@ucla.edu; 7 Department of Family Medicine, Adventist Health White Memorial Medical Center, Los Angeles, CA 90033, USA; FloresH1@ah.org

**Keywords:** diabetes, vegetarian diet, nutrition, metabolic syndrome, obesity, disparity

## Abstract

*Introduction:* The national rate of obesity in US Hispanic/Latinos exceeds all other major ethnic subgroups and represents an important health disparity. Plant-based diet interventions that emphasize whole plant foods with minimal processing and less refined grains and sugar have shown great promise in control of obesity, but there is a paucity of data translating this treatment effect to disparate populations. The objective of our study was to evaluate the efficacy of the Healthy Eating Lifestyle Program (HELP) for accomplishing weight management in a hospital-based, family centered, culturally tailored, plant-based diet intervention for Hispanic/Latino children who were overweight or obese. *Methods:* Our mixed methods evaluation included: (1) A one arm study to measure changes in body mass index (BMI) from pre- to post-intervention, and (2) A stakeholder analysis of the program staff. *Results:* For children ages 5–12 years who were overweight/obese, we found no evidence of excess weight gain evidenced by BMI Z scores (Z_post-pre_ = −0.02, *p* = 0.11). Among the parent/guardians who were overweight or obese, we found a decrease in BMI that was stronger in men (BMI _post-pre_ = −0.75 kg/m^2^, *p* = 0.01) than in women (BMI _post-pre_ = −0.12 kg/m^2^, *p* = 0.30). A program strength was the cultural tailoring of the plant-based diet choices. *Conclusions*: The evaluation raises the possibility that incorporating intervention components of HELP (plant-based food choices, family-based, cultural tailoring) into pediatric weight management can improve the standard of care.

## 1. Introduction

The 2016 National Health and Nutrition Examination Survey of the US reported a very high national prevalence of obesity in Hispanic/Latino adults ages 20 years and older (Hispanic/Latino (47%), Black (47%), Asian (13%), White (38%)) [1,2]. For Hispanic/Latino children, a similar disparity in the obesity trend was also found in the national data (26% Hispanic/Latino; 22% Black; 11% Asian; 14% White) [1,2]. Dr. Eduardo Sanchez, Chief Medical Officer for Prevention at the American Heart Association issued a statement on the disparity in obesity rates highlighting the need to link the public with “affordable, healthy, and nutritious foods, and fewer sugary drinks” [3].

Plant-based diet interventions that emphasize whole plant foods with minimal processing and less refined grains and added sugars have shown great promise in control of obesity and type 2 diabetes (T2DM). In a meta-analysis of randomized controlled trials of 15 plant-based diet interventions, Barnard et al. found that adherence to the diet for at least four weeks was associated with a mean weight loss of −3.4 kg [4]. In a systematic review of 9 randomized controlled trials of plant-based diets for T2DM patients, Toumpanikis et al. [5] reported that: (1) 8 out of 9 found a clinically important decrease in HbA_1c_ levels, and (2) 6 out of 9 reported reduction or discontinuation of diabetes medication. Taken together, we find convincing data from randomized controlled trials in the experimental setting that plant-based diet interventions can improve management of both obesity and T2DM. The next step in this research is the translation of plant-based diet treatment effects to high risk (i.e., for cardio-metabolic disease), disparate populations in the US such as Hispanic/Latinos.

For the purpose of systematically adapting a plant-based diet intervention for Hispanic/Latino patients at high risk for cardio-metabolic disease, our group at Loma Linda University (LLU) undertook a series of pilot studies [6,7,8,9] to study plant-based diet practices in Hispanic/Latino Adventists. The findings from these studies were used to further test whether such plant-based diet patterns could be applied to Hispanic/Latino adults receiving health care in a federally designated medically underserved region of Southern California. The rationale for studying Adventists is that due to faith-based recommendations of the Seventh-day Adventist Church (Protestant Christian Denomination), about 50% of the members are vegetarian, and are further encouraged to consume certain plant foods (i.e., legumes, nuts, whole grains) in place of animal products [10,11]. The US National Institutes of Health (NIH) have funded cohort studies at LLU that provide convincing evidence that a range of plant-based diet patterns (from semi-vegetarian to vegan) practiced by Adventists are associated with lower incidence of obesity and diabetes [12,13,14,15,16], and a longer life expectancy [17,18]. For example, in a national sample of 3475 US Hispanic/Latino Adventists [7] in the NIH-funded Adventist Health Study-2 (AHS-2), plant-based diet patterns were associated with significant percent decreases in body mass index (BMI) relative to patterns of frequently eating animal products: semi-vegetarians (−4.89 %, *p* = 0.011), pesco-vegetarians (−5.34%, *p* < 0.0001), vegetarians (−8.92%, *p* < 0.0001), vegan (−15.07%, *p* < 0.0001). In a Southern California sample of Mexican Adventists [6], the Adventist Multi-ethnic Nutrition Study (AMEN) study found that relative to omnivore diet patterns, plant-based diet patterns were associated with: (1) maintaining body mass index (BMI) in the recommended range of 19 to 25 kg/m^2^ (24.5 kg/m^2^ vs. 27.9 kg/m^2^, *p* = 0.006), and (2) significant decreases in waist circumference (34.8 vs. 37.5 in, *p* = 0.01), and fat mass (18.3 kg vs. 23.9 kg, *p* = 0.007). Lastly, LLU faculty adapted Adventist Health Study findings to the design of a pilot plant-based diet intervention trial that enrolled 32 Mexican T2DM patients from community clinics in a federally designated medically underserved area of Southern California (San Bernardino County) [8,9]. The intervention increased plant-based diet choices (without requiring strict vegetarianism) through a culturally tailored set of cookbooks, recipes, and cooking instruction designed by a Hispanic/Latino nutritionist [8,9]. After 6 months of follow-up, the investigators found a plant-based diet treatment effect (decrease in HbA_1C_ of 1.2%) [8] from the LLU pilot study that translates to a 21% decrease in diabetes-related deaths, 37% decrease in microvascular complications, and 14% decrease in risk of myocardial infarction [19].

For the scalability of interventions in disparate populations, the National Institute of Minority Health and Health Disparities proposes a conceptual framework that involves engaging multiple domains of influence over the lifespan (biological, behavioral, physical/built environment (i.e., household), sociocultural, and health care system (i.e., patient–provider)) [20]. For the present study, Loma Linda University partnered with Adventist Health-White Memorial Medical Center (AH-WMMC)—a health care organization operating in a medically underserved, East Los Angeles catchment area that is 90% Hispanic-Latino (85% Mexican). AH-WMMC has developed a successful model of culturally responsive care for their patient population [21,22], and this care model has been recently used to adapt and pilot test culturally tailored plant-based diet and physical activity interventions for Hispanic/Latino Patients. Specifically, the AH-WMMC Diabetes Education Office adapted a hospital-based, family centered intervention—the Healthy Eating Lifestyle Program (HELP) [23,24]—for use as a plant-based diet intervention for Hispanic/Latino pediatric patients with obesity and their families. HELP [23,24] was initially created by the Hospital Association of Southern California as a nutrition and exercise intervention for pediatric patients with obesity and their parents. During 2005–2007, 1135 children and 991 parents were enrolled in HELP through the Los Angeles Chronic Disease Management Consortium (California Hospital Medical Center (CHMC), Good Samaritan Hospital, Huntington Hospital). HELP participants decreased their BMI z-scores (*p* < 0.001) [23].

The overall aim of our mixed methods evaluation of the adapted HELP program (i.e., added plant-based diet focus) was to assess programmatic effectiveness and sustainability in reducing pediatric obesity by educating children ages 5–12 and their families about plant-based eating habits and physical activity (6 week educational phase). The specific aims are as follows: (1) To examine whether the HELP intervention prevented an increase in adiposity levels in obese/overweight children, (2) To examine whether the HELP intervention decreased adiposity in obese/overweight parents, (3) To determine strengths, weaknesses, opportunities, and threats to the HELP program effectiveness and viability through a stakeholder analysis of program staff.

## 2. Methods

In 2010, the Department of Family Medicine and Diabetes Education of Adventist Health White Memorial (AH-WMMC) Medical Center adapted and implemented HELP to be a culturally tailored plant-based diet and physical activity intervention for pediatric obesity in Hispanic/Latino families. At AH-WMMC, HELP was delivered through a highly successful model of culturally responsive care that was developed, implemented, and disseminated by AH-WMMC in 2008 [21,22]. We will describe the HELP Program, the qualitative evaluation methods of HELP, and its quantitative evaluation methods. Our program evaluation methods of the adapted HELP program received ethics approval from the Institutional Review Board of Adventist Health White Memorial Medical Center (#20191016) and the Institutional Review Board of Loma Linda University (#5190401).

### 2.1. Healthy Eating Lifestyle Program (HELP) Program

#### 2.1.1. HELP Implemented in the Context of a Culturally Competent Care Model Developed at White Memorial Medical Center

AH-WMMC created an innovative modular curriculum for training of providers [21], medical residents, and medical students in cultural competence that draws from the domains of cultural competence training from the American Association of Medical Colleges (AAMC) [21]: (1) Introduction/Key Concepts, (2) Bias Stereotyping, Culture, and Clinical Decision Making, (3) Health and Healthcare Disparities, (4) Cultural Competence in Patient Care, and (5) Cultural Competence and Community Action. The curriculum draws from theory-driven frameworks built upon cultural awareness, knowledge, skills, encounters, and proficiency [21]. A 2016 US Health Resources and Services Administration (HRSA) funded effort further disseminated AH-WMMC’s model for an East Los Angeles catchment area (90% Hispanic/Latino; 85% Mexican) to hospitals serving the South Los Angeles [22] catchment area that was also predominantly a population that was Hispanic/Latino. For the HELP program, this framework empowered the delivery of culturally tailored plant-based diet informational materials through a multidisciplinary provider staff (family medicine, nursing, diabetes educator, patient navigator) and through a common culturally responsive framework [21]. Over a 3 year interval, the model produced higher patient retention rates in diabetes clinics (88%) and nutrition referrals (12%) than would be expected in a medically underserved community [21].

#### 2.1.2. HELP Study Lifestyle Intervention

Study Population. Participants were enrolled in the HELP program using the following inclusion criteria: (1) a child ages 5–12 years, with body mass index (BMI) percentile ≥85% by CDC guidelines [25], (2) one parent/guardian must attend and participate in the workshop with the child participant, (3) no provider restriction for both child and parent to participate in the HELP diet and physical activity intervention. The primary method for recruitment was pediatric referral from the AH-WMMC service area. Flyers and advertisements in the AH-WMMC system, hospital magazine, and news media (English and Spanish Language television and print media) were used. Word of mouth and patient referrals were also accepted.

During 2010–2018, children and their family members were encouraged to participate in the program together. No family members were excluded. During this period, three hundred and forty-eight children participated in the program along with an additional 194 non-referred children (i.e., family members of referred children). Curriculum and data collection methods changed post 2015; hence, only participants enrolled in the HELP program from 2016–2018 are reported (52 referred children and 98 adults). Of the 52 children who were referred to the program due to meeting inclusion criteria, two were measured at < 85th percentile at the baseline visit but were not excluded from the program evaluation.

**Figure 1:**
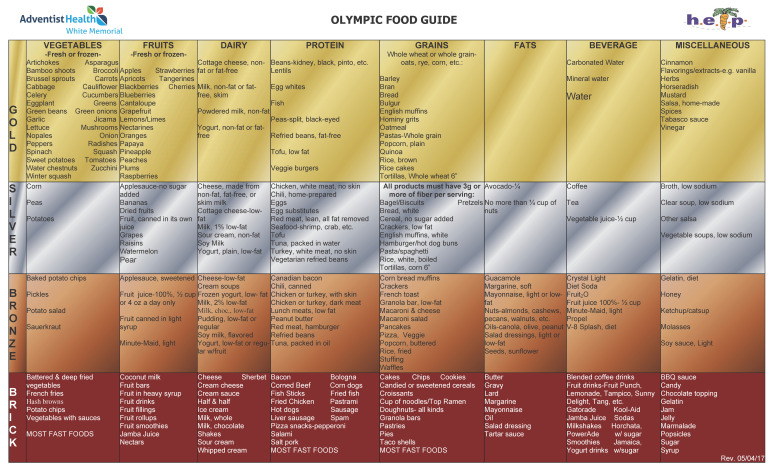
Tiered food guide for the Healthy Eating and Lifestyle Program (HELP) designed for Hispanic/Latino children ages 5–12 years who were overweight/obese.

The HELP Intervention was adapted to deliver culturally tailored plant-based diet guidance. The 5 month intervention included an intensive 6 week educational phase (6 lifestyle change modules) followed by a 3 month maintenance phase and program graduation. The dietary intervention consisted of cooking instruction and supermarket tours to implement a four tiered (gold, silver, bronze, brick) food guide to plant-based eating (Figure 1). The food guide is depicted in Figure 1, and shows that the highest tier (gold) involved eating whole plant foods with minimal processing (i.e., not battered, deep fried, heavily sauced, and/or fast food processing), and allowed a pesco-vegetarian pattern. Participants were coached on this tiered continuum and there were no strict vegetarian categories enforced (transitioning to gold-silver-bronze are all a “success”)—a method resonating with AHS-2 findings from Hispanic/Latinos that semi-vegetarian and pesco-vegetarian patterns have significant protective effects against obesity [7].

A further adaptation of the original HELP program consisted of interactive hands-on cooking classes conducted by expert bilingual staff (three registered dietitians/certified diabetes educators) from the local community. The programs focused on teaching culturally tailored plant-based nutrition: how to eat a healthy diet and how to cook plant-based healthy recipes. The interactive cooking classes allowed the participants from the community not only to learn about healthy eating, but experience it by participating in the cooking process, touring the supermarkets, and tasting the samples as part of the education. All of the program educational materials were specifically developed for the targeted community with language considerations.

Furthermore, all cooking demonstrations were designed with the target population in mind, where recipes were carefully aligned with the traditional fare of this community. The educators took special care to ensure that the recipes taught in the program included only those ingredients that were easily accessible in the local neighborhood markets, making the program recommendations easily attainable. This means participants did not have to alter their shopping habits and go out of their community to go grocery shopping. This is of particular importance as many in this community do not own a car, therefore having to drive or take a bus to a whole foods store would create an additional burden for this community. Instead, they could shop in the same neighborhood markets. The resources for healthier meals were easily available in their own community.

The physical activity intervention included a physical activity pyramid and pedometer measures that were designed to help parental/guardian participants monitor their and their child’s progress toward achieving pedometer goals (10,000 steps per day for the adult, and 13,000 steps per day for the child). For each parent–child dyad, height (by stadiometer) and weight was measured during program sessions (six times during the 5 month follow-up). A food and activity diary was recorded by the parent to monitor their and their child’s progress towards improvement as measured by the tiered food guide (Figure 1). The methods of monitoring physical activity and diet changed during 2010–2018 program implementation period and this precludes using these measures in the quantitative evaluation. Education (obesity knowledge, cooking instruction, and supermarket tours) was the major component of this intervention and was conducted by the Diabetes Program Manager, Certified Diabetes Educator, Dual Role Health Educator-Office Coordinator, Dual Role Health Educator-Patient Navigator, who were all bilingual and Hispanic/Latino.

The HELP Quantitative Outcome Measure is described. The goal of the HELP program is healthful lifestyle change. We tracked BMI by taking each child’s and each adult participant’s weight and height. HELP is a 6 consecutive week program with a 3 month break between week 6 (module 5) and graduation (module 6). We take their weight and height at either Orientation or module 1 (week 1 or 2), and at module 5.

### 2.2. Qualitative Measures and Analysis

Participants. During May 2018, semi-structured interviews were conducted with six key informants who possessed valuable insight about the HELP Program at White Memorial Medical Center (WMMC). These six key informants consisted of an interdisciplinary team (Diabetes Program Manager, Certified Diabetes Educator, Dual Role Health Educator-Office Coordinator, Dual Role Health Educator-Patient Navigator, Endocrinologist, Executive Chef). Four of the team members (Diabetes Program Manager, Certified Diabetes Educator, Dual Role Health Educator-Office Coordinator, Dual Role Health Educator-Patient Navigator) had specifically designed, culturally tailored, implemented, and modified the intervention during 2010 to the present. The two remaining team members (Endocrinology, Executive Chef for AH-WMMC) were chosen for their technical knowledge of diet and diabetes in patients in the catchment area.

Interviews. All participants provided written informed consent. The following four interview questions were developed by the Center for Health Research at School of Public Health, Loma Linda University to evaluate the sustainability of the HELP Program: (1) What are the strengths of the programs to introduce more plant-based eating into the diets of Hispanic/Latino obesity or T2DM patients being treated at AH-WMMC? We would like you to refer to cooking classes, supermarket tours, and diet counseling if possible, (2) What are the weaknesses of the programs to introduce more plant-based eating into the diets of Hispanic/Latino obesity or T2DM patients being treated atAH-WMMC? (3) What opportunities are present and need further development in the programs to introduce more plant-based eating into the diets Hispanic/Latino obesity or T2DM patients being treated at AH-WMMC? (4) What are the threats that exist to continuing development of the program to introduce more plant-based eating into the diets of Hispanic/Latino patients with obesity or T2DM being treated at AH-WMMC? The interviews with consented participants were held over the telephone during business hours and lasted 20–40 min. All interviews were digitally recorded (with participants’ consent) and transcribed verbatim. The interviewer reviewed the transcripts to ensure no content was lost during the transcription and to clarify any questions.

Qualitative Analysis. We used classic grounded theory to design a qualitative program evaluation that followed a minimally prescriptive approach that allowed themes to emerge from the data. Our rationale was that a family-based, culturally tailored, plant-based diet approach to treating pediatric patients with obesity is quite novel. Our qualitative evaluation of the HELP program will allow us to gain an in depth understanding of how this type of novel intervention can be implemented in a series of RCTs at the T2 to T4 level of intervention. The interview transcripts were entered into QSR International’s NVivo 11 qualitative data analysis software. Open and axial coding was used to analyze the data and identify emerging themes.

### 2.3. Quantitative Evaluation of the HELP Study

Outcomes. To assess the effect of the intervention on adiposity in the child and adult we conducted a quasi-experimental, one group evaluation to measures changes in BMI from baseline to week 6 (final week of education phase). The primary outcome was the modified CDC Z-score for a child’s BMI [26]. Since 2017, the CDC has provided a modified Z-score for children that helps tracking of children in the extremes of the BMI distribution [25]. A secondary outcome was adult BMI.

Analysis. Linear mixed models with main effects of time, gender, age, cohort year, gender by time interactions, and subject-level random intercepts were used to model the change in BMI in adults and BMI z-scores in children from baseline to post intervention. BMI and BMI z-score contrasts from these models were computed to specifically investigate the main effect of follow-up time (baseline to post intervention). Interactions of this main effect with gender were also considered in exploratory analyses. All analyses were done using SAS Software (version 9.4) (SAS Institute, Cary, NC, USA) and R version 3.1.1 (R Foundation for Statistical Computing, Vienna, Austria).

## 3. Results

### 3.1. Quantitative Study of Adiposity Outcomes in HELP Participants

Ninety-eight adults (87% females and 13% males; mean age = 41.2 years) and 52 children (58% females and 42% males; mean age = 9.3 years) were referred to the HELP program from 2016 to 2018. Ninety-three of the adults (98%) were classified as overweight or obese (BMI ≥ 25) when measured at baseline. Fifty of the children (96%) were classified as overweight or obese (BMI ≥ 85th percentile or BMI Z-score > 1.04 by CDC guidelines [25] yielded the same group) when measured at baseline.

For children who were ages 5–12 years and overweight or obese (*n* = 50), there was a slight decrease in modified BMI Z score (Z = −0.02, *p* = 0.11) from pre- to post-intervention (Z = 2.52 to Z = 2.50 in the girls; Z = 2.45 to Z = 2.43 in the boys). No significant effects were found for gender, age, cohort year, or a time by gender interaction.

**Figure 2:**
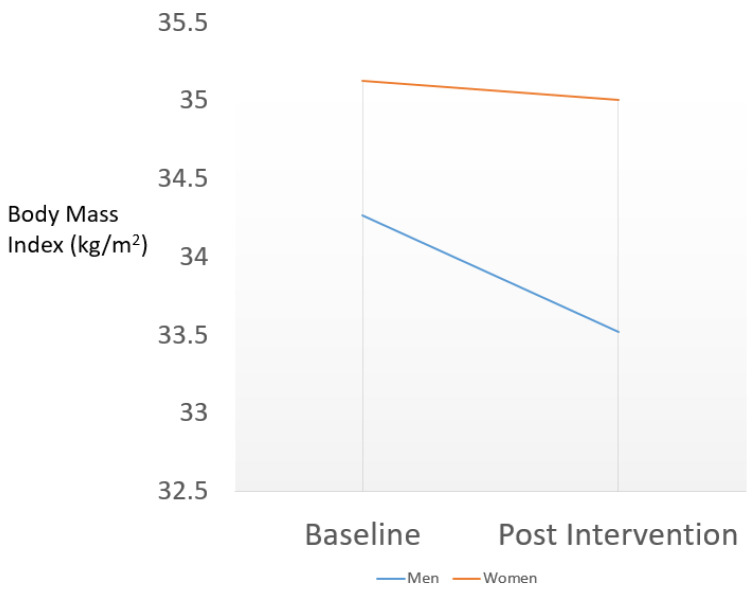
A baseline to post-intervention decrease in body mass index (BMI) among Hispanic/Latino Parent/Guardians in the Healthy Eating and Lifestyle Program who are overweight/obese (Body Mass Index ≥ 25) is computed from a linear mixed model with a significant (*p* = 0.047) interaction term for gender and follow-up time.

For adults who were overweight or obese (*n* = 93), there was a slight decrease in BMI (*p* = 0.06) from pre- to post-intervention of 0.21 kg/m^2^. No significant effects were found for age, gender, or cohort year, but we did find a significant interaction of time by gender (*p* = 0.047) that is depicted in Figure 2. The pre- to post-intervention decrease (34.27 to 33.52, men; 35.13 to 35.01, women) depicted in Figure 2 was much stronger in men (−0.75 kg/m^2^, 95% CI for the difference (−1.33 to −0.18), *p* = 0.01), as compared to women (−0.12 kg/m^2^, 95% CI for the difference (−0.36 to 0.11), *p* = 0.30).

### 3.2. Qualitative Study of HELP Study Providers

The six key informants provided insight into the strengths and weaknesses of the current programs as well as the opportunities for future development. Five key emerging themes were identified.

*Theme #1:* The surrounding community stands to benefit from nutrition programs at AH-WMMC.

One of the main emerging themes identified was that the surrounding community members stand to benefit from nutrition programs at AH-WMMC. This theme was evident across all six of the interviews. Participants referred to the socio-economic status and disease prevalence in the area, the prevalence of unhealthy nutritional habits, the positive response from the community members to the current program offering, and the openness of the community members to learn. As one of the key informants stated: “I feel, with experiences with other programs is that they really want to live healthier lives, they want healthy changes…we do have a community wanting and hungry to learn.”

*Subtheme #1a:* The patients served are largely a non-vegetarian community.

Half (three) of the participants highlighted that a plant-based diet is not common in this community. This presents challenges and opportunities of getting the community members to accept the plant-based diabetes education and to buy into a plant-based diet program considering how presently the community residents are not inclined to the vegetarian diet. Despite this, the key informants felt that the past experience with the program being well tailored to the community resulted in a positive response from community members: *“they were able to hear this is good for me, it’s easy to make, and not only it was good for me and it’s easy to make, but it doesn’t taste bad.”*

*Subtheme #1b:* Positive community response to the current program.

Four of the participants referred to the positive response from community members to the program being offered. One of them who is involved in coordinating the program shared positive feedback she received from the program participants, expressing gratitude: *“I get families telling us how thankful they are and how they benefited, how they didn’t know that what they were giving to their children was bad. How kids are now telling the parents how to eat better or not to buy certain products because that’s what they learned in class… Children are at a time in their life where the changes are going to be more permanent if we reach them, because they are more willing to learn.”*

*Theme #2:* Lack of awareness among community members about the program offered

Four of the participants shared that one of the challenges of the program was that the community members were not well informed on the availability of classes or on what diabetes education entails. Patient education on diabetes and obesity, while available on a one-on-one basis, may not reach every patient. Two participants stated that not all patients have the access to providers who know about the program and can provide a referral to take the diabetes education classes.

“Anybody can get that (diabetes education) as long as they have some sort of insurance coverage…Whichever patients have access to the educators, they will get the … education.”

One key informant who is among chief instructors for the programs expressed that classes have room to grow so more of the population would be able to benefit from them: *“I think maybe the only struggle would be to get the word out there, (get) people informed so we get bigger classes, a bigger population coming to get educated”.*

*Theme #3:* Lack of teen-focused programs

One of the key informants expressed a concern that there is not a next level of the child obesity program for this community, so that as the children transition in age, they could remain in the chronic disease prevention program and continue getting this support: *“I wish I had a teen program because ours (program) only runs from five to twelve…. I am a true believer of children’s programs because I think children are at a time in their life, for their changes are going to be more permanent if we reach them because they’re more willing to learn. The adults aren’t but children are more open and teens because there is a really, really great need that’s when a lot of the social the environment comes in and they really, really need that help.”*

*Theme #4:* Culturally Tailored Interactive Program Conducted by Competent Staff

Another emerging theme was that culturally tailored interactive programs conducted by competent staff, as is done at AH-WMMC, resulted in greater acceptance by the community. As one of the participants stated: *It’s not only good for me, it’s easy to cook, it tastes good, and I am able to purchase the items at my local market.”*

*Subtheme #4a:* Cultural Tailoring

Five of the key informants discussed in detail the cultural tailoring of the program and this appeared to be one of the largest reported strengths of the program. This means they could continue to enjoy familiar foods but now made with healthier ingredients: *“I did a lot of recipes that were familiar; they were Latin recipes they would bring to me, and I would turn them into healthier versions of their favorite recipe…I believe, that was well accepted.”*

*Subtheme #4b:* Tailored Educational Materials

In addition to cultural sensitivity, the educational materials developed for these programs reflected the low literacy level of the targeted population and included pictures, and as one of the participants stated: *“they are easy to follow”.*

*Subtheme #4c:* Competent personnel

Two of the key informants felt that having competent experienced staff (three bilingual registered dietitians, who are also certified diabetes educators, with prior experience in offering cooking classes and interactive health education) conducting the programs contributes greatly to the overall strengths of the HELP program.

*Theme #5:* Administrative and Financial Support is Necessary for the Success of Community Programs

Another important emerging theme that has the potential of becoming a barrier or the advantage to the diabetes prevention programs at AH-WMMC is related to the administrative and financial support of the programs.

*Subtheme #5a:* Leadership Support

Two of the respondents identified an important source of support provided by the hospital administration for the nutrition program: *“it’s the first time that the hospital has really taken on to fund our programs, in the past it was all done by grants…. The hospital is pretty much on board that this is an extremely important program and that diet definitely makes an impact for diabetes and pre-diabetes.”*. On the other hand, a third respondent felt less certain about internal funding for the future of the program and emphasized the importance of recognizing the importance and challenges of lifestyle interventions for disparate populations (i.e., large proportion Spanish monolingual with low literacy level or illiterate) when making decisions on resource allocation and marketing. For marketing the hospitals nutrition programs for pediatric obesity, the third respondent expressed the opinion that a more hospital-specific plan (as opposed to regional) would help them get the availability of the nutrition program better disseminated to the community being served.

*Subtheme #5b:* Financial Support

All six responded reported that a lack of consistent funding to the programs was a key weakness/threat to the programs. As community health programs do not bring in revenue, all the key informants expressed concern about their sustainability. Lack of funding for space rental and materials as well as inability to expand programs were among some of the reported concerns. One of the participants was encouraged about the hospital providing the program funding for the first time during this year, as in the past traditional grant funding resulted in lay-offs once the funding ran out. More diabetes educators are needed to sustain and expand quality programs, but the current staff’s concern is whether they will be able to keep who is there now: *“Can we have more diabetes educators, can we even keep the ones that we already have and then the ancillary staff that we need to run these types of programs”.*

Given the type of programs offered, two of the key informants referenced the use of grants for past funding and felt that there are multiple grants that could be sought after to sustain these programs in the future. The concern however, expressed at the same time, is that when the programs are funded by grants, it means the funding is limited to a time-period, and once it runs out, the programs may have to stop.

## 4. Discussion

Our findings from the HELP program indicate that for Hispanic/Latino children who were overweight/obese and lived in a medically underserved region of East Los Angeles, a culture-specific, family-tailored plant-based diet intervention prevented a significant increase in adiposity as measured by BMI Z-score. The family-based intervention also helped with weight management in their overweight/obese parents/caregivers, particularly in the males. Several aspects of HELP program findings reported here are noteworthy: (1) The plant-based diet intervention was not based on enforcing a strict adherence to vegetarianism, but instead promoted a tiered approach to following a pattern that emphasized incorporating whole plant foods with minimal processing (i.e., not battered, deep fried, heavily sauced, fast food processing), (2) The intervention allowed semi-vegetarian patterns, (3) The intervention improved weight management outcomes in both child and parent, (4) The plant-based diet intervention was administered by a multidisciplinary team that worked within a culturally responsive intervention model that made the intervention diet both culture-specific and family-tailored (i.e., participants bringing their family’s favorite recipes to the educators and being taught to prepare healthier versions of them).

Promoting Culture-specific Plant-based Diet Choices without using Dietary Pattern Labels (“Vegetarian”)

Our findings indicated that completion of the adapted HELP program that included a component that promoted increasing plant-based food choices (Figure 1) was an effective method for reducing excess adiposity in Hispanic/Latino families. We note that the highest tiers of the HELP diet promoted a more continuous spectrum of dietary improvement by increasing plant-based diet choices rather than requiring strict adherence to a vegetarian diet. Our findings are broadly consistent with the findings from 3475 Hispanic/Latino Seventh-day Adventists (Protestant Christian denomination encouraged to followed plant-based diet patterns) indicating that the significant decreases in adiposity observed for strict vegetarian and vegan patterns could also be achieved by adopting semi-vegetarian diet patterns [7].

From the standpoint of translation to a high risk disparate population for cardio-metabolic disease, we note that our findings move beyond promoting plant-based dietary pattern labels (i.e., vegetarian versus non-vegetarian) into the potentially more impactful domain of promoting culture-specific, familiar choices of whole plant foods with minimal processing from the tradition of pre-Columbian Mesoamerican Diet [27,28]. This was reinforced by our qualitative findings (subtheme 1a) in which providers voiced concerns that a strict vegetarian intervention would be too challenging to the cultural norms of Hispanic/Latino families in medically underserved areas.

Our qualitative work with the HELP staff identified the positive effect of a cultural tailoring of the choice of plant foods that was implemented through culturally responsive staff and family-tailoring of the recipes. This finding is consistent with findings from other interventions for Hispanic/Latinos. For example, Santiago-Torres et al. developed a “Traditional Mexican Diet Score” (MexD) that quantified a three sisters pattern (i.e., ↑corn-beans-vegetable, ↓refined grains/sugars) from dietary survey data from Mexican women and found the MexD score to be inversely related to inflammation (hsCRP) and insulin resistance [28]. In their subsequent crossover trial of healthy Mexican women, a Traditional Mexican Pre- Hispanic diet (↑corn-beans-cultural vegetable (i.e., nopales (cactus pads) and jicama)) produced significant decreases in insulin, insulin resistance, and IGFB3 as compared to a US diet [27]. In a pilot crossover trial in Baja, Mexico, Jimenez-Cruz et al. found that a low glycemic index Meso-American diet (↑pinto beans + whole meal bread, ↓refined grains/sugars) significantly decreased HbA_1c_ in T2DM patients [29]. Studies from South America report that vegan and semi-vegetarian Peruvian and Brazilian participants (following traditional cultural choices involving plant foods) do have lower rates of hypertension, dyslipidemia, and obesity as compared to omnivores [30].

Household/Family Tailoring of the Diet to affect the Household Context of the Patient: The “Familismo” Effect [31,32,33]

The “Familismo” Effect [31] in the Hispanic/Latino cultural context introduces the idea that “family comes first” and has been cited in the design of family-based, culturally tailored interventions. For example, an intervention that utilized a family-based diabetes intervention on behavioral and biological outcomes in Mexican adults indicated significant changes (*p* = 0.043) over time for behaviors such as self-managements in diet, exercise, and diabetes care compared to the control group (based on Diabetes Self-Care Activities Questionnaire) [33,34]. This study also found sustained self-management of general health, and a significant decrease in physician, regimen, and interpersonal distress [33,34].

T2DM patients who specified family participation as a motivating factor for making healthier lifestyle choices experienced a 1.4%–1.7% reduction in HbA1c in diabetes self-management studies [31]. Additional studies indicated a 0.41% drop in HbA1c and improvements in blood pressure and diabetes knowledge among study participants of a family-based diabetes intervention involving Hispanic Adults [27]. Data from this study underscored the importance of family involvement with findings that showed that BMI and diabetes knowledge also improved significantly among the non-diabetic family members who were involved in the intervention [27].

### Limitations

We note the major limitation of our evaluation is the quasi-experimental design in a small sample where we do not have a control arm to isolate the dietary treatment effect. The findings herein need investigation in a randomized controlled trial that has a sample size that allows consideration of individual and family effects. Additionally, we note the issues of validity and reliability of the diet and physical activity measures.

## 5. Conclusions

Findings from HELP provide preliminary evidence that a culture-specific, family tailored intervention delivered in the context of culturally responsive care by a health care organization can be an effective intervention for pediatric and adult obesity in Hispanic/Latino families in a medically underserved region. HELP intervened on multiple domains across the life course of the NIMHD Research Framework: Biological (caregiver-child interaction), Behavioral (Family Functioning, Household Environment, Family Norms), Built Environment (Supermarket Shopping), and Health Care System (Provider–Patient) [20]. Further investigation of the efficacy of this intervention in a randomized control trial is the next step in this research.
